# Utilizing image and caption information for biomedical document classification

**DOI:** 10.1093/bioinformatics/btab331

**Published:** 2021-07-12

**Authors:** Pengyuan Li, Xiangying Jiang, Gongbo Zhang, Juan Trelles Trabucco, Daniela Raciti, Cynthia Smith, Martin Ringwald, G Elisabeta Marai, Cecilia Arighi, Hagit Shatkay

**Affiliations:** Department of Computer and Information Sciences, University of Delaware, Newark, DE 19716, USA; Department of Computer and Information Sciences, University of Delaware, Newark, DE 19716, USA; Amazon, Seattle, WA 98109, USA; Department of Computer and Information Sciences, University of Delaware, Newark, DE 19716, USA; Google, Mountain View, CA 94043, USA; Department of Computer Science, The University of Illinois at Chicago, Chicago, IL 60612, USA; Division of Biology and Biological Engineering, California Institute of Technology, Pasadena, CA 91125, USA; The Jackson Laboratory, Bar Harbor, ME 04609, USA; The Jackson Laboratory, Bar Harbor, ME 04609, USA; Department of Computer Science, The University of Illinois at Chicago, Chicago, IL 60612, USA; Department of Computer and Information Sciences, University of Delaware, Newark, DE 19716, USA; Department of Computer and Information Sciences, University of Delaware, Newark, DE 19716, USA

## Abstract

**Motivation:**

Biomedical research findings are typically disseminated through publications. To simplify access to domain-specific knowledge while supporting the research community, several biomedical databases devote significant effort to manual curation of the literature—a labor intensive process. The first step toward biocuration requires identifying articles relevant to the specific area on which the database focuses. Thus, automatically identifying publications relevant to a specific topic within a large volume of publications is an important task toward expediting the biocuration process and, in turn, biomedical research. Current methods focus on textual contents, typically extracted from the title-and-abstract. Notably, images and captions are often used in publications to convey pivotal evidence about processes, experiments and results.

**Results:**

We present a new document classification scheme, using both image and caption information, in addition to titles-and-abstracts. To use the image information, we introduce a new image representation, namely *Figure-word*, based on class labels of subfigures. We use word embeddings for representing captions and titles-and-abstracts. To utilize all three types of information, we introduce two information integration methods. The first combines Figure-words and textual features obtained from captions and titles-and-abstracts into a single larger vector for document representation; the second employs a meta-classification scheme. Our experiments and results demonstrate the usefulness of the newly proposed Figure-words for representing images. Moreover, the results showcase the value of Figure-words, captions and titles-and-abstracts in providing complementary information for document classification; these three sources of information when combined, lead to an overall improved classification performance.

**Availability and implementation:**

Source code and the list of PMIDs of the publications in our datasets are available upon request.

## 1 Introduction

Biomedical research findings are typically reported via publications. To simplify access to domain-specific knowledge, while supporting the research community, several biomedical databases [e.g. UniProt (Bateman *et al.*, 2021), BioGRID (Chatr-Aryamontri *et al.*, 2017), Wormbase (Harris *et al.*, 2020) and MGI (Blake *et al.*, 2021)] invest significant effort in expert curation of the literature. The first step in the biocuration process is to identify articles that are relevant to a specific area on which the biomedical databases focus. For example, biocurators at the Jackson Laboratory’s Gene Expression Database (GXD) identify publications relevant to gene expression during mouse development (Finger *et al.*, 2017). Manually selecting biomedical publications in such focus areas is often too labor-intensive and slow for effectively detecting all and only the relevant articles within a large volume of published literature. As such, automatically identifying publications relevant to a specific topic is an important task toward expediting biocuration and, in turn, biomedical research.

The vast majority of current methods for categorization of biomedical documents focus on textual contents which are typically extracted from the title and the abstract of the publication. Several supervised learning methods, including Support Vector Machines (SVMs) (Garcia *et al.*, 2015), Decision Trees (Almeida *et al.*, 2014) and Neural Networks (Burns *et al.*, 2019; Fergadis *et al.*, 2018), have been applied and studied to build document classifiers. Burns *et al.* (2019) investigated the application of several word embedding methods using different neural network configurations for identifying scientific literature containing information about molecular interaction. Rule-based methods have also been proposed for document classification (Hu *et al.*, 2005; Karystianis *et al.*, 2017). For instance, to identify epidemiological publications, Karystianis *et al.* (2017) developed a set of rules based on syntactical patterns observed from the training documents. Notably, current methods utilize only textual information, while important research processes and experimental results are often reported via images and their captions in publications.

Figures and captions convey fundamental, essential information in biomedical documents. As such, there is a growing interest in storing, browsing and in utilizing images and their respective captions as a source of knowledge. In particular, biomedical databases are beginning to store and to display images as evidence for a variety of processes and for experimental results (Finger *et al.*, 2017; Liechti *et al.*, 2017). Notably, most current biomedical publications are stored as Portable Document Format (PDF). An essential step toward making use of images is the extraction of figures and captions from the PDF files of publications. Several systems have been developed for identifying and extracting figures and captions from scientific documents (Clark *et al.*, 2016; Li *et al.*, 2019).

**Fig. 1.**
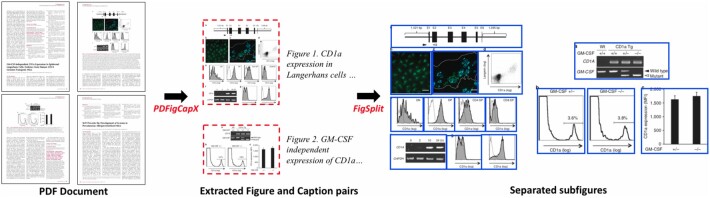
An example of our pipeline for figures, subfigures and captions extraction. The original PDF document (Kobayashi *et al.*, 2012) is shown on the left. Figures (dashed red boxes) and captions are first extracted from the document using PDFigCapX. Figures then be further separated into subfigures (solid blue boxes) using FigSplit

Another obstacle toward utilizing biomedical images is the abundance of compound figures comprising multiple panels (see e.g. Fig. 1), where each panel often conveys a distinct information type obtained via one of several possible diverse modalities. For instance, both *graphs* and *gel* images may appear side-by-side as panels in a single figure providing evidence for similar or for distinct findings. In order to utilize the information from individual subfigures, it is essential to segment compound images into their constituent panels. Identifying compound figures and their constituent panels is a topic of much research (Chhatkuli *et al.*, 2013; Santosh *et al.*, 2015), including our own (Li *et al.*, 2018).

Image captions have been shown effective for document classification in several studies (Burns *et al.*, 2019; Jiang *et al.*, 2017, 2020; Regev *et al.*, 2002). For instance, Burns *et al.* (2019) compared classification performance under different information sources, when identifying publications containing molecular interaction information, relevant to the IntAct Molecular Interaction database (Kerrien *et al.*, 2012). Their experiments showed that a classifier utilizing figure captions outperformed classifiers using information from either the title-and-abstract, MeSH terms, body text or figure description from the body text. Our group is one of the first to use image content information for biomedical document classification (Ma *et al.*, 2015; Shatkay *et al.*, 2006). Shatkay *et al.* (2006) first proposed to use the class label of figures (such as: *line chart*, *gel electrophoresis* and *fluorescence microscopy*) as image features to identify publications that are relevant to the Gene Ontology annotation task performed by the Mouse Genome Informatics at the Jackson Laboratory. A more recent work from our group integrates information extracted from figures using Optical Character Recognition with text information for identifying documents that are relevant to cis-regulatory modules (Ma *et al.*, 2015). However, none of the current methods use image captions and image contents together. Thus, we aim to integrate information from both image contents and their respective captions, in addition to titles-and-abstracts, toward improving biomedical document classification.

Here we introduce a new scheme that utilizes information from images, captions and title-and-abstracts toward improved biomedical document classification. To do this, we first extract figures, subfigures/panels and captions from the documents. In order to represent figures within biomedical documents, we propose a new image representation, namely, *Figure-word* that encodes the combination of different types of panels within a figure. An image taxonomy is also introduced and used to train a classifier for categorizing the extracted panels. For handling text-contents, we employ word embeddings (Moen and Ananiadou, 2013), for both caption-based and title-and-abstract-based document representations. To utilize all three types of information sources (images, captions and titles-and-abstracts), two information integration methods are introduced. The first combines Figure-words and features obtained from captions and title-and-abstract into a single larger vector for document representation; while the second employs a meta- classification scheme.

The rest of the paper presents the details of our method, and demonstrates its effectiveness through a series of experiments. Section 2 describes the complete framework of our method; Section 3 presents experiments and results, assessing its performance; Section 4 discusses and analyzes the results, while Section 5 concludes and outlines directions for future work.

## 2 Methods

Our goal is to identify biomedical documents that are relevant to a specific domain by utilizing images and captions along with titles-and-abstracts. To do that, we first extract figures and their captions from the PDF files of biomedical documents, by employing the parsing tool that we have developed—and is now publicly available—PDFigCapX (https://www.eecis.udel.edu/∼compbio/PDFigCapX) (Li *et al.*, 2019). As many of the extracted figures are compound images comprising multiple panels, we also separate such figures into their constituent panels, using our previously developed FigSplit (https://www.eecis.udel.edu/∼compbio/FigSplit) system (Li *et al.*, 2018) for compound image separation.

To represent images within biomedical documents, we first introduce an image taxonomy comprising 12 categories, which serves as framework for classifying biomedical figures. Next, we train an image classifier to categorize the extracted panels. We introduce a new image representation, namely, *Figure-word*, which encodes the combination of different types of panels in a figure, and use it to generate an image-based representation of each document, $d_{\text{IMG}}$. Word embeddings, which convert a word to a numerical vector of a fixed number of dimensions have been prevalently used for text representation (Mikolov *et al.*, 2013). As such, we use word embeddings pre-trained over a corpus of biomedical articles to generate, for each document *d*, its caption-based representation, $d_{\text{CAP}}$, as well as its title-and-abstract-based representation, $d_{\text{TA}}$. We introduce two information integration methods to utilize the information from images and captions, in addition to titles-and-abstracts. The first method concatenates the representations $d_{\text{IMG}},\;d_{\text{CAP}}$ and $d_{\text{TA}}$ into a single larger vector for representing each document, *d*. The second is a meta-classification approach, combining the output of base classifiers that were trained separately over images, captions and titles-and-abstracts to train the final document classifier.

### 2.1 Extracting figures, subfigures and captions from biomedical documents

To utilize image information, we first extract images and their corresponding captions from the PDF file of biomedical publications. Extracting figures and captions is not a simple task due to the complex and diverse layout of biomedical publications and the variations in figure structure, texture and contents. To extract images and their captions from biomedical publications, which are primarily stored as PDF files, we use PDFigCapX (Li *et al.*, 2019). Unlike other methods that extract figures by handling raw encoded contents of PDF documents, PDFigCapX begins by separating text from graphical contents, utilizing layout information to detect and disambiguate figures and captions. Files containing the figures and their associated captions are produced as output.

The vast majority of the extracted figures are compound images consisting of multiple panels. In order to utilize image information from each individual panel, we use our FigSplit tool (Li *et al.*, 2018), segmenting compound images into their constituent panels. Unlike other methods that segment images using gaps between panels, FigSplit identifies panels based on Connected Component Analysis. It also overcomes the common issues of over- and under-segmentation by evaluating and self-correcting candidate segmentations that are likely to be inaccurate.

Both systems, PDFigCapX and FigSplit, were tested on existing and on newly assembled datasets, demonstrating robustness and significant improvement compared to other state-of-the-art methods (Li *et al.*, 2018, 2019). Figure 1 shows an example of our pipeline for extracting figures, subfigures and captions from biomedical publications. The input to our pipeline is the original PDF document shown on the left. By using PDFigCapX, figure and caption pairs are extracted. The extracted images are shown in red dashed boxes. By applying FigSplit, compound images are split into their constituent panels—each shown within a solid blue box on the right.

### 2.2 Image-based document representation

Figures in biomedical publications are typically used to show the process and results of experiments. Different types of images are used to report certain types of experiments. For example, gel images are typically used in pull-down assays (Orchard *et al.*, 2012). Class labels of figures have been shown useful for document representation in biomedical document classification in our previous work (Shatkay *et al.*, 2006). As discussed in Section 2.1, the majority of figures within biomedical publications are compound images. Building upon our previous idea, we introduce here a new method to represent figures within documents based on class labels of their constituent panels here.

**Fig. 2.**
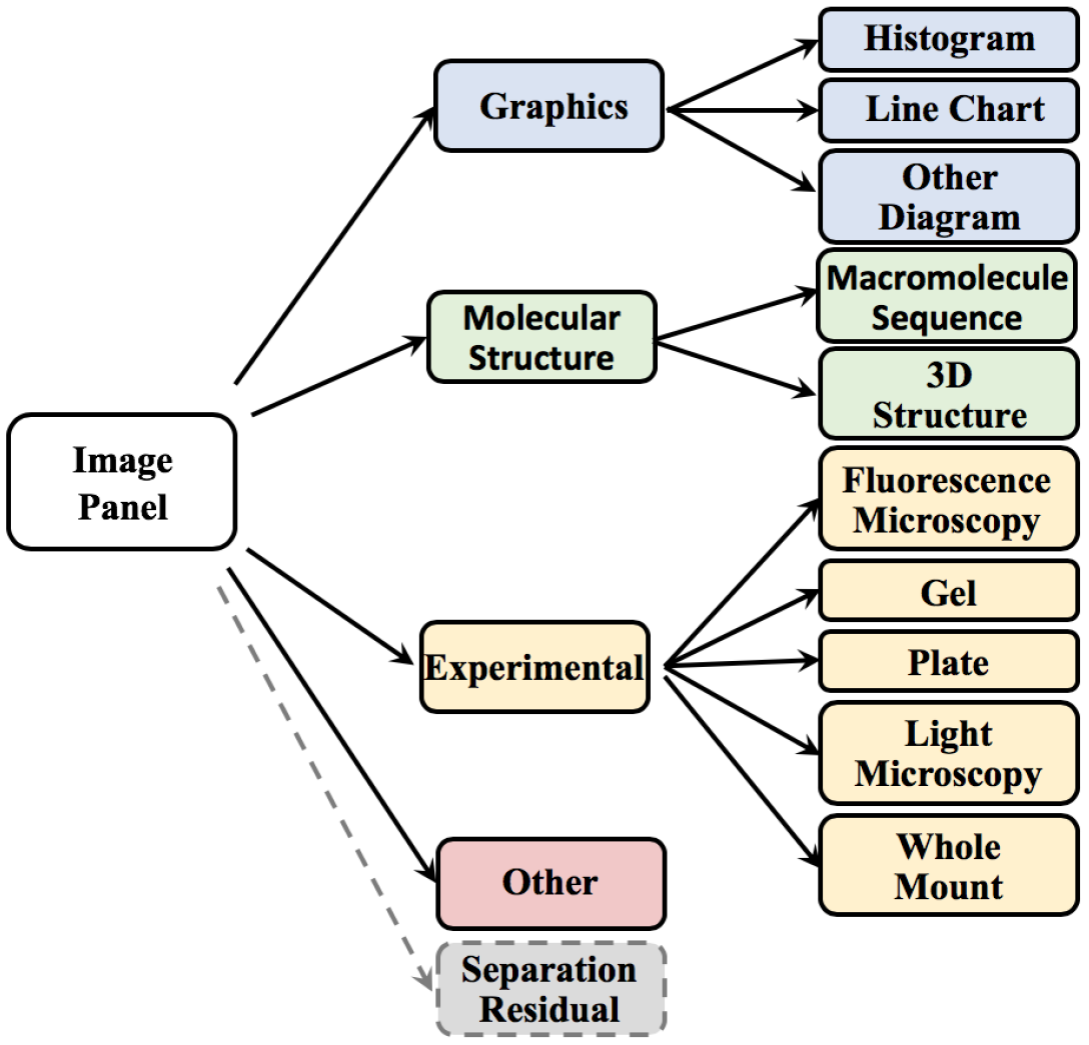
The image taxonomy used for panel classification

While several image taxonomies were proposed for classifying biomedical images (De Herrera *et al.*, 2016; Lopez *et al.*, 2013; Shatkay *et al.*, 2006), as no standard exists, we extend the image taxonomy previously proposed by our group, through collaboration with GXD (Finger *et al.*, 2017), Protein Information Resource (Wu *et al.*, 2003) and WormBase (Harris *et al.*, 2020), as shown in Figure 2. At the top level, images are classified into *Graphics, Molecular Structure, Experimental* and *Other* images. At the second level, *Graphics* are classified into *Histogram, Line Chart* and *Other Diagram*. *Molecular Structure* images are classified into *Macromolecule Sequence* and *3D Structure* images. *Experimental* images are further classified into *Fluorescence Microscopy, Light Microscopy, Whole Mount, Gel* and *Plate* images. We also note that figure legends or margins are sometimes over-separated from their original images by the compound image separation process, thus forming individual panels. We refer to such panels formed by over-segmentation of compound images as separation residuals. As these residuals do not belong to any of the informative taxonomy’s classes, we augment our taxonomy with a *separation residual* class.

To automatically assign class label to individual panels, we build an image classifier. A pre-trained Convolutional Neural Network, VGG16 (Andrearczyk and M**ü**ller, 2018; Simonyan and Zisserman, 2015), is used for image classification. To train the classifier, we use the annotated image dataset that was introduced by Lopez *et al.* (2013) based on the Molecular INTeraction database dataset (Licata *et al.*, 2012). The image dataset consists of 34 876 pre-labeled panels; its statistics are shown in Table 1. In addition, a set of 500 labeled whole mount images were provided by GXD. Trained and tested via a 5-fold cross validation, the classifier demonstrates 87.89% accuracy.

**Table 1.** Distribution of image types included in our experiments, based on the image dataset introduced by Lopez *et al.* (2013)

	Histogram	Line chart	Other diagram	Macromolecule sequence	3D structure	Fluorescence microscopy	Gel/blot	Plate	Light microscopy	Other	Separation residual
No. of panels	4270	2664	3536	499	1424	5714	14865	508	1156	130	110

**Fig. 3.**
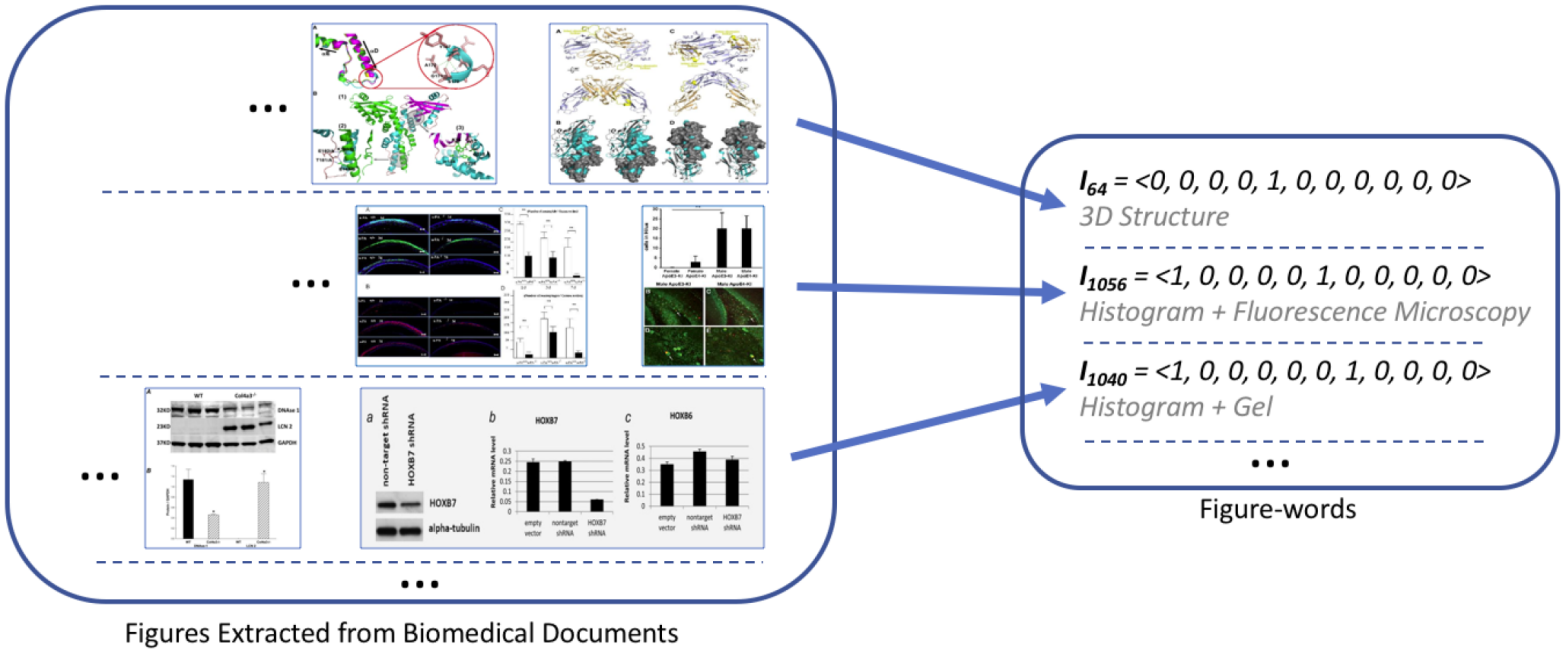
The process for converting figures into their corresponding Figure-words. The set of figures extracted from the biomedical documents is shown on the left. The corresponding Figure-words along with their vector representations indicating the types of comprising panels, are shown on the right. The images on the left are taken from (Li *et al.*, 2008, Fig. 4; Qiu and Dhe-Paganon, 2011, Fig. 6; Sugioka *et al.*, 2014, Fig.3; Leung *et al.*, 2012, Fig. 9; Heinonen *et al.*, 2015, Fig. 4; Dai *et al.*, 2012, Fig. 3)

Once the class label of each panel is obtained, we represent each figure as an 11-dimensional binary vector $<c_{1},c_{2},\ldots ,c_{i},\ldots ,c_{11}>$, where *c_i_* is 1 if a panel from class *i* is present in the figure and 0 otherwise. For instance, if the figure comprises only histograms and fluorescence microscopy panels (panels of type1 and type6 respectively), its corresponding vector is: <1, 0, 0, 0, 0, 1, 0, 0, 0, 0, 0>. We refer to each such vector as a Figure-word. Figure 3 shows the process for converting figures into their corresponding Figure-words. As the number of classes in our image-taxonomy is 11, the total number of possible Figure-words in our vocabulary is 2^11^ (2048). A document *d*, in turn, is represented as a vector $d_{\text{IMG}}=<I_{1},I_{2},\ldots ,I_{i},\ldots ,I_{n}>$, where $n=2^{11}$ and *I_i_* (1≤i≤2048) is 1 if the *i*th Figure-word appears in the document *d*, 0 otherwise.

### 2.3 Caption-based document representation

Captions associated with figures provide another important source of information for biomedical document classification. In order to make use of captions, we employ a standard preprocessing procedure that includes named-entity recognition (NER), stemming and stop-words removal as we have done in our earlier work (Jiang *et al.*, 2017, 2020). For NER, we first identify all gene, disease, chemical, species, mutation and cell-line concepts using PubTator, which is widely used for annotations of biomedical concepts (Wei *et al.*, 2019). We then substitute each of the identified concepts by its respective generic terms ‘gene’, ‘disease’, ‘chemical’, ‘species’, ‘mutation’ or ‘cell-line’. We also stem words using the Porter stemmer and remove standard stop words (Porter, 1980; Canese and Weis, 2013). The concatenated captions are used as the caption-based document representation.

Word embeddings map words to vectors of a fixed dimension so that words appearing in similar contexts are mapped to similar vectors. Such a vectorized representation has been widely used for text classification (Moen and Ananiadou, 2013), and more recently for biomedical named entity recognition and biomedical relation extraction (Lee *et al.*, 2020). A word embedding model (Moen and Ananiadou, 2013) has been pre-trained specifically on a biomedical corpus, which consists of PubMed titles-and-abstracts and PubMed Central full text articles by employing the word2vec tool (Mikolov *et al.*, 2013). We use such embeddings to represent the concatenated captions. Each word, *w_i_*, within the concatenated captions is converted to a word embedding vector $\vec{w_{i}}$ whose dimensionality is 200. The document *d* is then represented as a 200-dimentional vector $d_{\text{CAP}}$, calculated as $d_{\text{CAP}}=\frac{1}{n}(\vec{w_{1}}+\vec{w_{2}}+\cdots +\vec{w_{n}})$, where *n* is the total number of distinct words in the concatenated captions, and $\vec{w_{i}}$ is the embedding vector of the *i*th distinct word.

### 2.4 Title-and-abstract-based document representation

The title and the abstract of articles are the text components most often used for biomedical document classification. To represent a document based on those, we first obtain the title-and-abstract of each publication. Similar to the steps described in Section 2.3, we employ a standard preprocessing procedure that includes named-entity recognition, stemming and stop-words removal to each title-and-abstract. The same word embeddings described in Section 2.3 are employed to convert each word *w_i_* in the preprocessed text to a word embedding vector $\vec{w_{i}}$. The document *d* is then represented as a 200-dimensional vector, denoted as $d_{\text{TA}}$, by calculating the mean of embedding vectors that are associated with words in the preprocessed text.

### 2.5 Information integration for document classification

So far, we have introduced document representations based on images ($d_{\text{IMG}}$), captions ($d_{\text{CAP}}$) and title-and-abstracts ($d_{\text{TA}}$). Next, we present two schemes for integrating the information stemming from these three sources.

**(1) Integration via concatenated vectors**

Under this scheme, to represent a document *d*, we simply concatenate the vectors $d_{\text{IMG}},\;d_{\text{CAP}}$ and $d_{\text{TA}}$ into a single vector $d_{\text{ALL}}$, thus utilizing the information obtained from images, captions and titles-and-abstracts. Recall that the value of an entry in the $d_{\text{IMG}}$ vector is either 1 or 0 which indicates whether or not a Figure-word appears in a document, while the value of an entry in $d_{\text{CAP}}$ or $d_{\text{TA}}$ is obtained by calculating the mean of embedding vectors converted from words in a caption or a title-and-abstract. There is no specific limit on the range of embedding vectors. Therefore, the values of such entries are at different scales from that of entries in $d_{\text{IMG}}$. As such, we standardize each feature within $d_{\text{ALL}}$ by rescaling the features such that they have a mean of 0 and a standard deviation of 1. For classifying the documents, we conducted experiments with several classification schemes, including Random Forests, Na_**ï**_ve Bayes (not shown here) and SVMs. As SVMs have been commonly used for both image and text classification, and have shown the best performance in this context (Holzinger *et al.*, 2014; Simpson *et al.*, 2015), we use SVM as the model for classifying the final resulting document-vectors, and denote this SVM classifier *CombV*.

**(2) Integration via meta-classification**

Another approach we propose toward integrating the multiple types of information is to employ a meta-classification scheme, which combines results obtained from multiple base classifiers into a single classification output. To do that, we first separately train three base classifiers $C_{\text{IMG}},\;C_{\text{CAP}}$ and $C_{\text{TA}}$ using the representations of images $\left(d_{\text{IMG}}\right)$, captions ($d_{\text{CAP}}$) and titles-and-abstracts ($d_{\text{TA}}$). By applying a base classifier to a document *d*, we obtain the class label *L* and the probability *P* of document *d* to be assigned to the relevant class. Each document *d* is then represented as a 6-dimensional vector $d_{\text{CombC}}=<L_{\text{IMG}},P_{\text{IMG}},L_{\text{CAP}},P_{\text{CAP}},L_{\text{TA}},P_{\text{TA}}>$. This representation is then used for training another classifier, referred to as meta-classifier, denoted as *CombC*, which assigns the final class label to each document. Similar to the concatenation-based integration, we use SVMs both as base classifiers and as the ultimate classifier in the meta-classification.

## 3 Experiments and results

To evaluate our method we conduct two sets of experiments. The first aims to compare the classification performance obtained when using only a single type of information to represent documents versus the performance when employing a representation that combines all three types of information. The classifiers using the representations of Figure-words, captions and titles-and-abstracts, are denoted as $C_{\text{IMG}},\;C_{\text{CAP}}$ and $C_{\text{TA}}$, respectively.

In the second set of experiments, we compare the performance of our system when utilizing a representation that combines images, captions and titles-and-abstracts to the performance attained by three state-of-the-art systems. The first system to which we compare is a random forest-based method (*RF_CAP_*) developed by Jiang *et al.* (2017) for identifying publications that are relevant to GXD. This classifier uses features extracted from the title-and-abstract and from caption text. The second is a convolutional neural network triage system (*CNN*${}_{\mathrm{BiLSTM}}$) presented by Burns *et al.* (2019) for identifying publications containing information about molecular interactions. The *CNN_BiLSTM_* classifier uses captions only. The third is a hierarchical recurrent neural network classification system (*HRNN*) developed by Fergadis *et al.* (2018) for identifying publications containing information about protein-protein interactions affected by genetic mutations; and uses title-and-abstract only. We compare all three systems using the code provided by their respective authors. For comparison, we run five complete rounds of 5-fold cross validation with 5 different 5-way partitions of the dataset. All experiments are conducted on a DELL machine that uses an Intel Core i7-6700 processor, an Nvidia GTX 1070 GPU, 8 GB of RAM and 256 GB of SSD.

### 3.1 Datasets and evaluation

In our experiments, we use two datasets for which we have the ground-truth class-labels. The first dataset, denoted *GXD_2000_*, is a subset of the dataset used by Jiang *et al.* (2017), who is also the developer of *RF_CAP_*. The original dataset is a collection of 58 362 publications (provided as PDF), curated by the Jackson Lab’s GXD throughout the years 2012–2016. As a first test of our method, we selected at random 1000 relevant and 1000 irrelevant documents from these publications, while retaining the same distribution of publication-years as in the larger GXD dataset. In order to use figures and captions, we first apply PDFigCapX to the *GXD_2000_* dataset. 8939 figures and 8594 captions are extracted from the relevant publications, while 8414 figures and 8042 captions are extracted from the irrelevant publications. We note that the number of figures extracted exceeds that of the captions, as some pages display figures (or parts of figures) without associated captions. FigSplit is then applied to separate compound figures into their constituent panels, resulting in 60 194 individual panels extracted from the 8939 figures associated with relevant publications and 41 015 panels obtained from the 8414 figures associated with the irrelevant publications.

The second dataset used in our experiments, denoted *DSP*, was introduced by Burns *et al.* (2019) for testing their system *CNN_BiLSTM_*. It comprises 537 publications relevant to molecular interactions and 451 irrelevant ones spanning the year range 1996-2017. Only publications for which PDF files are available are used in our experiments. As such, out of the 537 relevant publications only 534 are used, while out of the 451 irrelevant ones only 448 are retained, as their PDF files were available for download online. We then apply PDFigCapX and FigSplit to identify and extract figures, captions and constituent panels of extracted figures. From the 534 relevant publications, 3975 figures, 3912 captions and 21 421 panels are extracted, while 2928 figures, 2832 captions and 14 224 panels are extracted from the 448 irrelevant ones. Table 2 shows the statistics for these two datasets.

**Table 2.** The number of figures, captions and panels identified and extracted from publications in the datasets used in our experiments

Datasets	Classes	No. of docs	No. of figures	No. of captions	No. of panels
*GXD_2000_*	Relevant	1000	8939	8594	60 194
	Irrelevant	1000	8414	8042	41 015
*DSP*	Relevant	534	3975	3912	21 421
	Irrelevant	448	2928	2832	14 224

The total time for PDFigCapX to process the *GXD_2000_* dataset of 2000 publications is about 5.9 h (10.60 s per document, wall clock) where the average document contains 8.7 figures, 8.3 captions and is 7.2MB in size. It takes about 4.3 h (0.83 s per image, wall-clock) for FigSplit to process all extracted figures where on average 50.6 panels are extracted from each publication within the *GXD_2000_* dataset. Over the *DSP* dataset, PDFigCapX takes about 2.4 h (8.69 s per document, wall-clock) to process all 982 publications where the average document contains 7.0 figures, 6.9 captions and the average file size is 2.8MB. FigSplit takes about 1.6 h (0.78 s per image, wall-clock) to process all extracted figures where on average 36.3 panels are extracted from each publication in the *DSP* dataset.

To evaluate the document classification performance, we use standard measures, *Precision*, *Recall* and *F-score* defined as:

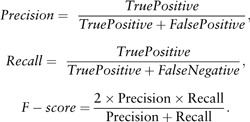


### 3.2 Results

Table 3 presents the classification performance attained when using only a single type of information to represent documents, along with the performance when employing a representation that combines all three types of information in our first set of experiments. The second to fourth columns in the table show results from experiments over the *GXD_2000_* dataset. Our classifier $C_{\text{IMG}}$, utilizing the Figure-words alone, attained 80.5% precision, 77.0% recall and 78.7% F-score. By using information from the caption alone, our classifier $C_{\text{CAP}}$ attained 88.6% in precision, 87.1% in recall and 87.8% in F-score. A similar performance (87.5% precision, 87.7% recall and 87.6% F-score) is attained by the classifier $C_{\text{TA}}$ when titles-and-abstracts are used to represent documents. Notably, a significant improvement is attained by using the representation that combines information from images, captions and titles-and-abstracts. By employing the meta-classification scheme, our classifier *CombC* attained 88.7% precision, 89.9% recall and 89.3% F-score. Our classifier *CombV* attained the highest performance of 89.4% precision, 91.0% recall and 90.2% F-score when the concatenated vectors are used for document representation. The performance attained by using the integrated information is statistically significantly higher than the performance attained based on the Figure-words, the captions or the titles-and-abstracts alone (*P*** **<** **0.01, two-sample *t*-test for all measures).

**Table 3.** Classification performance attained by using information from images ($C_{\text{IMG}}$), captions ($C_{\text{CAP}}$), the title-and-abstract ($C_{\text{TA}}$), concatenated vectors from all three types (CombV), and by using the meta-classifier (CombC). The highest values attained are shown in boldface. Standard deviations are shown in parentheses.

	*GXD_2000_*			*DSP*		
Classifiers	Precision	Recall	F-score	Precision	Recall	F-score
$C_{\text{IMG}}$	0.805 (.021)	0.770 (.026)	0.787 (.021)	0.679 (.018)	0.768 (.026)	0.731 (.026)
$C_{\text{CAP}}$	0.886 (.027)	0.871 (.032)	0.878 (.021)	0.804 (.024)	0.809 (.034)	0.806 (.021)
$C_{\text{TA}}$	0.875 (.021)	0.877 (.015)	0.876 (.013)	0.790 (.023)	0.807 (.023)	0.798 (.015)
*CombC*	0.887 (.019)	0.899 (.025)	0.893 (.008)	0.822 (.032)	0.826 (.044)	0.823 (.020)
*CombV*	**0.894** (.019)	**0.910** (.017)	**0.902** (.008)	**0.831** (.014)	**0.834** (.031)	**0.832** (.019)

The three rightmost columns in Table 3 present the results attained over the *DSP* dataset. When the Figure-words are used for document representation, our classifier $C_{\text{IMG}}$ attained 69.7% precision, 76.8% recall and 73.1% F-score. The classifier $C_{\text{CAP}}$ attained 80.4% precision, 80.9% recall and 80.6% F-score. A similar performance (79.0% precision, 80.7% recall and 79.8% F-score) is attained by $C_{\text{TA}}$ when titles-and-abstracts are used for document representation. Again, a significant improvement is attained by using the integrated information. We attained 82.2% precision, 82.6% recall and 82.3% F-score when the meta-classification scheme is applied. The highest performance of 83.1% precision, 83.4% recall and 83.2% F-score is attained when the concatenated vectors are used to represent documents. The performance attained by classifiers that integrate information from images, captions and titles-and-abstracts is statistically significantly higher than the performance attained by classifiers that are based on single information source (*P*** **<** **0.01, two-sample t-test for all measures). Our results demonstrate that our information integration schemes indeed improve biomedical document classification.

Table 4 compares the performance of our classifier CombV to that attained by the three other state-of-the-art systems, *RF_CAP_*, *CNN_BiLSTM_* and *HRNN*. Over the *GXD_2000_* dataset, *RF_CAP_* attained 82.9% recall, 87.8% F-score and the highest precision of 93.4%. *CNN_BiLSTM_* achieved 87.6% precision, 85.0% recall and 86.2% F-score, while *HRNN* attained 85.5% in precision, 87.5% in recall and 86.4% in F-score. While the precision (89.4%) attained by our classifier is slightly lower than that reached by *RF_CAP_*, our classifier *CombV* attained the highest recall of 91.0% and the highest F-score of 90.2% over the *GXD_2000_* dataset. Notably, recall is often viewed as more important than precision for biomedical document curation (Fang *et al.*, 2012; M**ü**ller *et al.*, 2004). Moreover, the differences between the results obtained by our system and those attained by *RF_CAP_*, *CNN_BiLSTM_* and *HRNN* are statistically significant (*P*** **<** **0.001, two-sample *t*-test). The three rightmost columns in Table 4 presents the results attained over the *DSP* dataset. *RF_CAP_* achieved 79.8% precision, 80.9% recall and 79.8% F-score, while *HRNN* attained 75.4% precision, 81.8% recall and an F-score of 78.3%. The *CNN_BiLSTM_* system, whose author introduced the *DSP* dataset itself, reached 82.0% precision, 79.6% recall and 80.9% F-score. Our classifier attained the highest performance of 83.1% precision, 83.4% recall and 83.2% F-score over the *DSP* dataset. Moreover, the differences between the results obtained by our classifier and those attained by other state-of-the-art systems are statistically significant (*P*** **<** **0.001, two-sample *t*-test).

**Table 4.** Classification performance Comparison with other state-of-the-art systems. The highest values attained are shown in boldface. Standard deviations are shown in parentheses.

	*GXD_2000_*			*DSP*		
Classifiers	Precision	Recall	F-score	Precision	Recall	F-score
*RF_CAP_*	**0.934** (.017)	0.829 (.037)	0.878 (.018)	0.798 (.068)	0.809 (.057)	0.798 (.030)
*CNN_BiLSTM_*	0.876 (.028)	0.850 (.031)	0.862 (.013)	0.820 (.023)	0.796 (.030)	0.809 (.018)
*HRNN*	0.856 (.044)	0.875 (.033)	0.864 (.010)	0.754 (.032)	0.818 (.054)	0.783 (.017)
*CombV*	0.894 (.019)	**0.910** (.017)	**0.902** (.008)	**0.831** (.014)	**0.834** (.031)	**0.832** (.019)

## 4 Discussion

Notably, Figure-words provide important information for document classification. The image-based classifier, $C_{\text{IMG}}$, which uses the newly proposed Figure-words alone for document representation attained 80.5% precision, 77.0% recall and 78.7% F-score over the *GXD_2000_* dataset, while attaining 69.7% precision, 76.8% recall and 73.1% F-score when applied to the *DSP* dataset (Table 3). Moreover, Figure-words provide information distinct from that captured by captions or by titles-and-abstracts. Of the *GXD_2000_* dataset, 71 relevant publications (7.1% of the relevant publications) were correctly identified by $C_{\text{IMG}}$, but incorrectly classified by $C_{\text{CAP}}$, while 71 publications were correctly identified by $C_{\text{IMG}}$, but incorrectly classified by $C_{\text{TA}}$. Of the *DSP* dataset, 59 relevant publications (11.0% of the relevant data) were correctly identified by $C_{\text{IMG}}$, but incorrectly classified by $C_{\text{CAP}}$, while 56 publications (10.5% of the relevant data) were correctly identified by $C_{\text{IMG}}$, but incorrectly classified by $C_{\text{TA}}$.

Another noteworthy point is that captions provide distinct information from that provided by titles-and-abstracts for document classification. As indicated in Section 3.2, the performances attained using a classifier based on captions or titles-and-abstracts alone ($C_{\text{CAP}}$ and $C_{\text{TA}}$, respectively) are similar over both the *GXD_2000_* and the *DSP* datasets. However, the relevant publications identified by classifiers $C_{\text{CAP}}$ and $C_{\text{TA}}$ are quite different. Of the *GXD_2000_* dataset, 60 relevant publications (6.0% of the relevant ones) were correctly identified by $C_{\text{CAP}}$, but incorrectly classified by $C_{\text{TA}}$, while 66 distinct publications were correctly identified by $C_{\text{TA}}$, but incorrectly classified by $C_{\text{CAP}}$. Of the *DSP* dataset, 38 relevant publications (7.1% of the relevant data) were correctly identified by $C_{\text{CAP}}$, but incorrectly classified by $C_{\text{TA}}$, while 37 distinct publications were correctly identified by $C_{\text{TA}}$, but incorrectly classified by $C_{\text{CAP}}$. By their very nature, titles-and-abstracts form a high-level summary of an entire study, while captions present details of experimental processes and results. This difference is reflected in the vocabulary of titles-and-abstracts versus that of captions. For instance, words such as *anterior, WT, dorsal, embryo, green, lateral* and *mount*, are commonly found in captions of publications relevant to GXD when describing gene expression experiments in mouse embryos. As such, captions provide information that is distinct from that provided through titles-and-abstracts, thus supporting more effective classification.

**Fig. 4.**
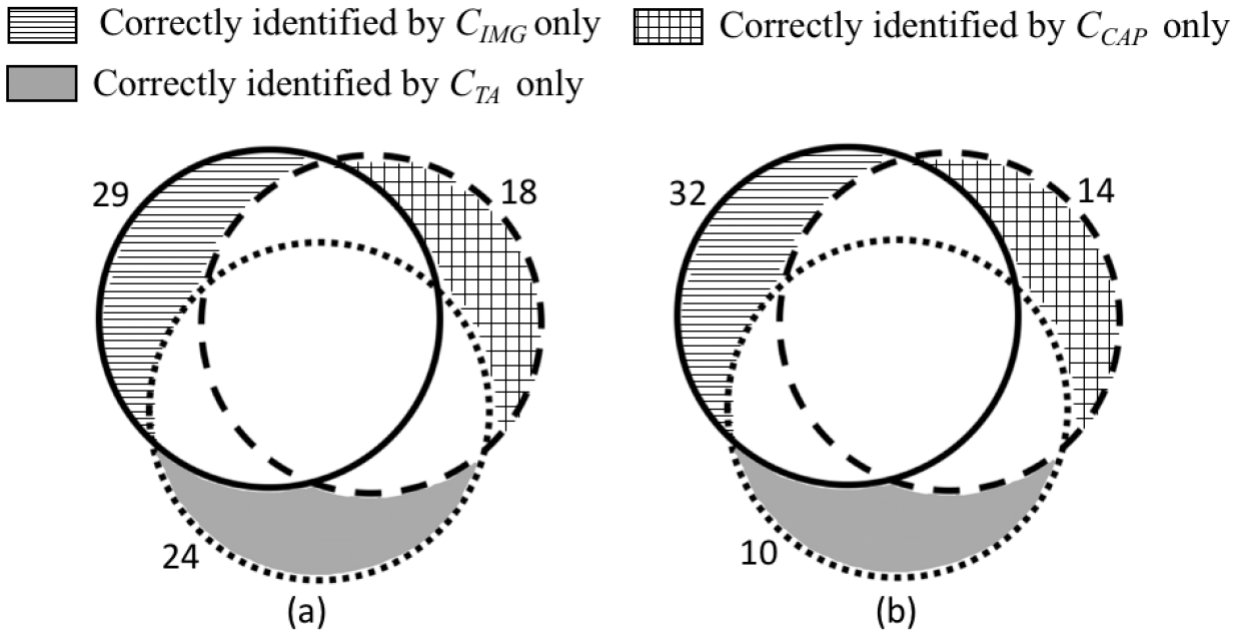
Comparison of classification results between the classifiers $C_{\text{IMG}},\;C_{\text{CAP}}$ and $C_{\text{TA}}$. The solid circle corresponds to the relevant publications that are correctly identified by classifier $C_{\text{IMG}}$. The relevant publications correctly classified by $C_{\text{CAP}}$ are indicated as a dashed circle, while those correctly identified by $C_{\text{TA}}$ are shown as a dotted circle. The region marked by horizontal stripes indicates the relevant publications identified by classifier $C_{\text{IMG}}$ only. The region marked by grid pattern corresponds to the relevant publications classified by $C_{\text{CAP}}$ only, while the region shown in solid gray indicates those identified by $C_{\text{TA}}$ only. (**a**) The comparison over the *GXD*_2000_ dataset. (**b**) The comparison over the *DSP* dataset

Figure 4 illustrates the respective classification results attained by the classifiers $C_{\text{IMG}}$, $C_{\text{CAP}}$ and $C_{\text{TA}}$. Of the *GXD_2000_* dataset, 18 relevant publications are identified only by $C_{\text{CAP}}$, while 24 relevant ones are identified only by $C_{\text{TA}}$. Notably, 29 relevant publications can only be identified by $C_{\text{IMG}}$ using Figure-words for document representation. Of the *DSP* dataset, classifier $C_{\text{IMG}}$ identified 32 relevant publications that are distinct from those identified by $C_{\text{CAP}}$ and $C_{\text{TA}}$. These findings strongly suggest that the three data sources, namely, Figure-words, captions and titles-and-abstracts provide distinct and complementary information for document classification.

To better understand the contribution of Figure-words to improved classification, we identify the most distinguishing Figure-words by ranking them based on the Z-score Test (Myers *et al.*, 1993), as we have done before for identifying distinguishing text terms (Jiang *et al.*, 2017, 2020). Table 5a shows the top-5 scoring Figure-words, along with their occurrence frequency in the relevant and in the irrelevant publications of the *GXD_2000_* dataset. There is a significant difference between the Figure-word distribution in relevant publications and their distribution in irrelevant ones. For instance, there are 1339 images consisting of fluorescence alone in relevant publications of the *GXD_2000_*, while only 437 such images in the irrelevant publications. Similarly, Table 5b shows that the distinguishing Figure-words identified with respect to the *DSP* dataset, demonstrate a clear difference in Figure-word distribution between relevant publications and irrelevant ones. Therefore, we believe that our newly proposed Figure-words have much potential for improving biomedical document classification.

**Table 5.** Top scoring Figure-words that contribute to the document classification task. The left most column in each table shows the Figure-words and their corresponding vectors indicating the types of comprising panels. The other two columns show the occurrence frequencies of corresponding figures in the relevant and in the irrelevant dataset, respectively. (a) Top Figure-words identified over the GXD2000 dataset. (b) Top Figure-words identified over the DSP dataset.

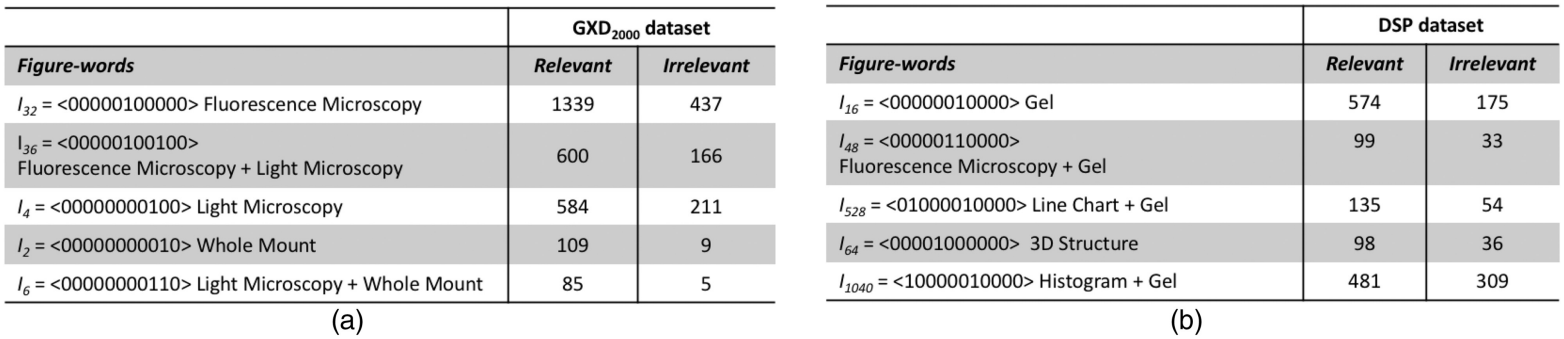	

**Fig. 5.**
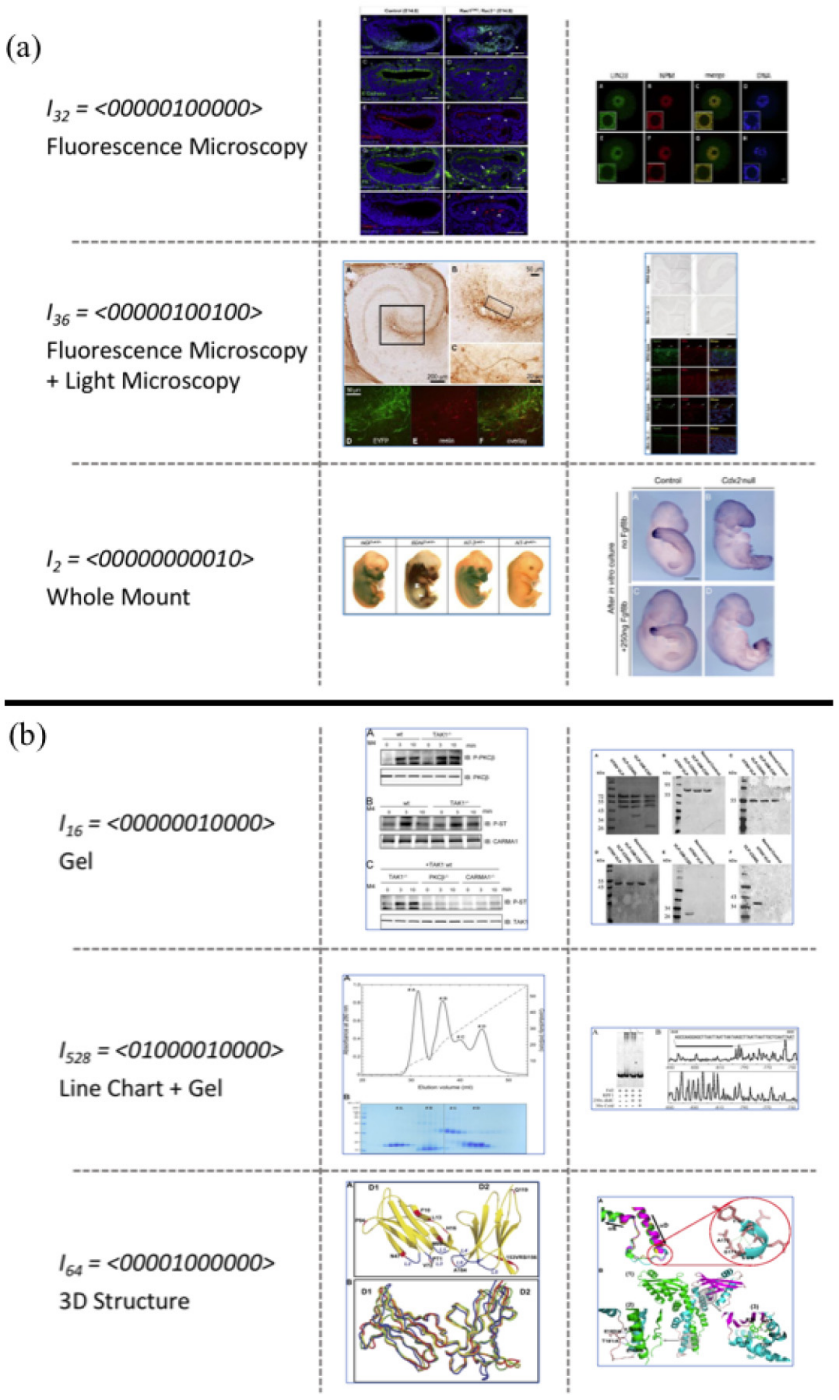
Examples of images for top scoring Figure-words. The leftmost column shows Figure-words along with their vector representations and their respective combination of panel types. The rest of the columns show examples of corresponding images. (**a**) Image examples from the *GXD*_2000_ dataset. Original images are taken from (Vogt *et al.*, 2012, Fig. 1; Grimsley-Myers *et al.*, 2012, Fig. 7; Yamaguchi *et al.*, 2014, Fig. 5; Quattrocolo & Maccaferri, 2014, Fig. 1; Liu *et al.*, 2012, Fig. 6; Rooijen *et al.*, 2012, Fig. S3). (**b**) Image examples from the *DSP* dataset. Original images are taken from (Shinohara *et al.*, 2005, Fig. 3; Cheng *et al.*, 2016, Fig. 4; Mysling *et al.*, 2016, Fig. 1; Yoshida *et al.*, 2014, Fig. 3; Graef *et al.*, 2009, Fig. 6; Li *et al.*, 2008, Fig. 4)

Figure 5 shows examples of Figure-words. Notably, the top scoring Figure-words also correspond to images that are typically found in distinct biomedical experiments. For instance, fluorescence microscopy images often appear in publications relevant to the GXD as this is a common imaging technique for visualizing gene expression. As curators at the GXD focus on mouse embryo studies, Figure-words containing embryonic whole-mount images are also indicative of documents that are likely relevant to GXD. Similarly, Co-inmmunoprecipitation, and Pull Down experiments are commonly used in studies relevant to molecular interactions, thus Figure-words corresponding to the gel/blot images are important for document classification over the *DSP* dataset (Fig. 5b). As such, our newly proposed Figure-words compactly account for and convey certain types of biomedical experiments. Experimental evidence is important for identifying relevant biomedical documents (Burns *et al.*, 2018; Han *et al.*, 2006), thus our Figure-words can contribute much informative evidence to the document classification task.

As discussed above, Figure-words, captions and titles-and-abstracts provide complementary information for document classification. A significant improvement is obtained by using the representation comprising all three sources. In our experiments, we attained statistically significantly improved performance by employing the meta-classification scheme *CombC* as well as by employing the classifier, *CombV*, where concatenated vectors are used for document representation, as compared to classification based on the title-and-abstracts, the captions and the Figure-words alone.

In the second set of experiments when our system is compared against three state-of-the-art systems, over the *GXD_2000_* dataset, two classifiers (*CNN_BiLSTM_*, *HRNN*) utilizing only a type of information source attained similar performance. The *RF_CAP_* classifier which uses features extracted from combined title-and-abstract and caption text, indeed outperforms the systems that use just a single type of information. Our method, which integrates the information from Figure-words, captions and titles-and-abstracts attained the highest recall and F-score over the *GXD_2000_* dataset. When applied to the *DSP* dataset, our method attained the highest score across all measures. These results demonstrate that Figure-words provide information distinct from that provided by titles-and-abstracts and by captions for supporting classification, and also prove the effectiveness of the integration methods that we introduced.

While our method indeed improves classification performance, there is still room for improvement, especially for the image taxonomy. In the work reported here, we utilized the image taxonomy consisting of 11 categories, as there is no unique standard image taxonomy for categorizing biomedical research images yet. A more comprehensive taxonomy has the potential to support a more informative Figure-words vocabulary and as such improve the overall document classification results. In our future work, we plan to expand and refine the image taxonomy we employ for categorizing biomedical research images. We are already in the process of applying our classification scheme to a larger dataset, namely the COVID-19 open research dataset comprising more than 50 000 articles (Chen *et al.*, 2020; Wang *et al.*, 2020), and plan to further apply it to the complete GXD dataset of more than 58 000 publications used by Jiang *et al.* (2017).

As our newly proposed Figure-words correspond to distinct images used in certain types of biomedical experiments, our image representation method can help biocurators identify images according to their experiment types. For example, a biocurator may want to identify images used in yeast two-hybrid experiments based on the images used to describe such experiments. We will also investigate the usage of our image representation for other tasks, such as biomedical image classification.

## 5 Conclusion

We presented a new scheme for identifying biomedical documents that are relevant to a certain domain, by using information derived from both *images* and *captions*, as well as from *titles-and-abstracts*. To do so, we first employed a pipeline for processing biomedical documents, comprising two parts: PDFigCapX that extracts figures with their captions from documents, and FigSplit for splitting the extracted compound figures into constituent panels, to biomedical documents.

A new image representation, Figure-word that encodes the combination of different types of panels is proposed for representing figures within documents. For captions and titles-and-abstracts, word embeddings are employed to represent documents as vectors. To utilize both image and caption information, in addition to titles-and-abstracts, we introduced two information integration methods. The first concatenates Figure-words and features obtained from captions and titles-and-abstracts into a single larger vector for document representation; the second employs a meta-classification scheme. Our experiments demonstrate the effectiveness of the newly proposed Figure-words for representing images. Moreover, classification performance is improved through the integration of information from all three sources, namely, images, captions and titles-and-abstracts.

As part of future work, we plan to build a more comprehensive taxonomy for refining image classification and improving the document classification performance. It is noteworthy that the newly proposed Figure-words correspond to certain distinct types of images used in reporting biomedical experiments. We will investigate the potential usage of Figure-words for other tasks, such as biomedical image classification.

## Funding

This work was partially supported by National Institutes of Health (NIH)/National Library of Medicine (NLM) awards [R56LM011354A and R01LM012527]; NIH/National Institute of Child Health and Human Development (NICHD) award [P41 HD062499 to M.R.].

*Conflict of Interest*: none declared.
