# Corrigendum to: Utilizing image and caption information for biomedical document classification

**DOI:** 10.1093/bioinformatics/btab568

**Published:** 2021-08-28

**Authors:** Pengyuan Li, Xiangying Jiang, Gongbo Zhang, Juan Trelles Trabucco, Daniela Raciti, Cynthia Smith, Martin Ringwald, G Elisabeta Marai, Cecilia Arighi, Hagit Shatkay

*Bioinformatics* (2021), Volume 37(Suppl1), i468–i476, doi:10.1093/bioinformatics/btab331

In the original published version, the formulae for *Precision* and *Recall* (Page i472, Right Column, Lines 32–33) were erroneously provided as:

$$\mathrm{Precision}=\;\frac{\mathrm{TruePositive}}{\mathrm{TruePositive}+\mathrm{FalseNegative}},$$

 

$$\mathrm{Recall}=\;\frac{\mathrm{TruePositive}}{\mathrm{TruePositive}+\mathrm{FalsePositive}}$$

The correct formulae that were consistently used throughout the experiments are as follows:

$$\mathrm{Precision}=\;\frac{\mathrm{TruePositive}}{\mathrm{TruePositive}+\mathrm{FalsePositive}},$$

 

$$\mathrm{Recall}=\;\frac{\mathrm{TruePositive}}{\mathrm{TruePositive}+\mathrm{FalseNegative}}$$

The error is thus only in mis-typing the formulae themselves; not in the actual calculations of the precision and the recall used throughout the paper.

As such, the other parts of the manuscript – specifically the experimental results reported, all stand as they appear in the original publication, and are not impacted by this correction.

